# Predicting anesthetic infusion events using machine learning

**DOI:** 10.1038/s41598-021-03112-2

**Published:** 2021-12-08

**Authors:** Naoki Miyaguchi, Koh Takeuchi, Hisashi Kashima, Mizuki Morita, Hiroshi Morimatsu

**Affiliations:** 1 Department of Intelligence Science and Technology, Kyoto University, Kyoto, 6068501 Japan; 2 Department of Biomedical Informatics, Okayama University, Okayama, 7008558 Japan; 3 Department of Anesthesiology and Resuscitology, Okayama University, Okayama, 7008558 Japan

**Keywords:** Computer science, Medical research, Mathematics and computing

## Abstract

Recently, research has been conducted to automatically control anesthesia using machine learning, with the aim of alleviating the shortage of anesthesiologists. In this study, we address the problem of predicting decisions made by anesthesiologists during surgery using machine learning; specifically, we formulate a decision making problem by increasing the flow rate at each time point in the continuous administration of analgesic remifentanil as a supervised binary classification problem. The experiments were conducted to evaluate the prediction performance using six machine learning models: logistic regression, support vector machine, random forest, LightGBM, artificial neural network, and long short-term memory (LSTM), using 210 case data collected during actual surgeries. The results demonstrated that when predicting the future increase in flow rate of remifentanil after 1 min, the model using LSTM was able to predict with scores of 0.659 for sensitivity, 0.732 for specificity, and 0.753 for ROC-AUC; this demonstrates the potential to predict the decisions made by anesthesiologists using machine learning. Furthermore, we examined the importance and contribution of the features of each model using Shapley additive explanations—a method for interpreting predictions made by machine learning models. The trends indicated by the results were partially consistent with known clinical findings.

## Introduction

In the field of medicine, anesthesiologists are responsible for pain and palliative cares, with particular emphasis on the biological management of patients during surgeries. To provide safe medical care to patients, anesthesiologists need to physically stay with patients for a long period of time during operations, and provide appropriate treatments before and after the operations; otherwise, there might be a risk that patients will feel pain during surgeries or have residual symptoms. However, recently, owing to a serious shortage of anesthesiologists, it has become difficult for anesthesiologists to be fully involved in all surgeries, and the increasing burden on anesthesiologists has become a major issue.

In addition to the issues of workload, the management of anesthesia is extremely difficult because the attributes of patients and the vital signs observed during surgeries are complex; moreover, these attributes must be considered before performing the appropriate procedure on patients. Therefore, these complexities increase the possibility that human errors may occur in the procedure, and it is especially difficult for inexperienced anesthesiologists to perform procedures of sufficient quality.

The widespread use of electronic medical records recently has made it possible to collect and store considerable amounts of anesthesia record data. However, the amount of data continues to increase annually; because the volume of these data is so significant, there are few opportunities for anesthesiologists directly use these data. Therefore, to effectively use the collected data, data analysis using machine learning technology, which has made remarkable progress recently, is becoming increasingly important.

Against this background, recently, research on the applications of machine learning to anesthesiology has been conducted. For example, research is being conducted to support decision making by anesthesiologists during surgery, including on topics such as risk prediction during surgery and the prediction of the depth of anesthesia. Among these techniques, risk prediction during surgery and bispectral index (BIS) prediction are topics that have been actively pursued.

By predicting risks before a surgery takes place, anesthesiologists can take actions at an early stage, which benefits patients by reducing sequelae and improving survival rates. One of the most frequently addressed issues in risk prediction is hypotension prediction. Intraoperative hypotension is known to be associated with postoperative acute kidney injury and noncardiac postoperative myocardial injury, and the risk of these conditions is quite high^1^. A typical problem setting for predicting the risk of hypotension is the classification problem of predicting whether hypotension will occur in some future period of time for a particular patient. There have been many similar studies on predicting the occurrence of hypotension^2–8^. In another study, instead of predicting the occurrence of hypotension, a bidirectional recurrent neural network was used to make real-time predictions of future blood pressure, 3 min into the future^9^. Aside from hypotension, risk prediction for arrhythmia and hypoxemia has also been studied, although there have been fewer studies on these topics. With respect to arrhythmia, Yoon et al. considered a model to predict tachycardia^10^. Moreover, Solomon et al. predicted bradycardia at three time points: the start of anesthesia induction, the start of surgery, and 30 min after the start of surgery, and examined the relationship between these data and hypotension^11^. Lundberg et al. used gradient boosting and Lasso to predict the occurrence of hypoxemia, and developed an interpretable model capable of elucidating which features contribute to the prediction of hypoxemia and under what circumstances^12^.

The advantage of predicting BIS in advance is that it helps to predict the effect of the sedative drug, propofol, thus allowing the dosage of the drug to be changed according to the predicted value of BIS. Olivier et al. showed that a lazy learning method outperforms a conventional linear model in predicting BIS, and argued that machine learning methods can extract useful information from anesthesia history^13^. Furthermore, Sakuma et al. used a recurrent neural network (RNN) to predict BIS, and conducted experiments via simulation, while considering a system to control anesthesia^14^. In another study, Lee et al. built a model using long short-term memory (LSTM) to predict BIS based on the history of propofol and remifentanil use as well as basic information such as the patient’s gender, age, height, and weight^15^. They also showed that the neural network-based method has better performance in predicting BIS compared to the response surface model, which considers the pharmacokinetic and pharmacodynamic (PK/PD) modeling^16^.

As introduced above, there have been various studies on the application of machine learning to anesthesiology, but their purpose was mainly to support anesthesiologists during operations. Although these studies have contributed to reducing risks and improving performance during operations, they have not directly solved the problem of the shortage of anesthesiologists. Therefore, what is of greater necessity is research on the automatic control of anesthesia, which, when realized, will solve the problem concerning shortage of anesthesiologists, while reducing the burden on anesthesiologists and decreasing human errors.

There is a rich history of research on the control of anesthesia. One of the earliest studies used arterial pressure as an index to control isoflurane using fuzzy logic^17,18^. Later, when the BIS became the standard measure of patient sedation, a closed-loop control method was proposed with the goal of maintaining a constant BIS^19^. Specifically, there has been significant research on propofol control, and various methods have been proposed, including methods using target controlled infusion with PK/PD models and methods using proportional integral derivative, as described in the review by Ilyas et al^20^. Recent studies have proposed reinforcement learning methods that consider PK/PD models and use deep learning for reinforcement learning^21,22^.

**Figure 1:**
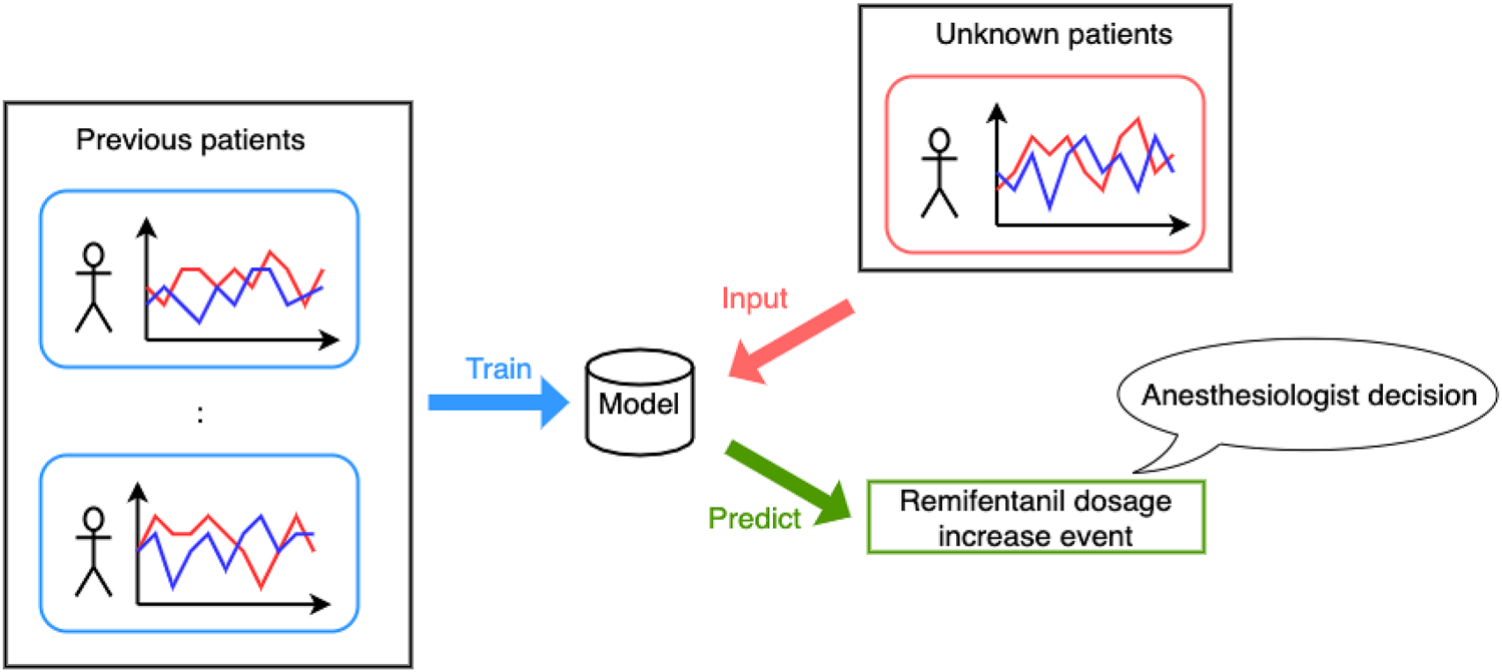
Overview of the decision prediction system considered in this study. The model is trained using supervised learning to predict future anesthetic decisions for unknown patients.

In most existing research, drug administration is controlled with the goal of maintaining the effect of anesthetics at a certain predetermined value, based on basic patient information and observed values such as BIS. However, the actual decision making of anesthesiologists must consider vital signs other than indicators. Therefore, using only the effect of anesthetics for control ignores various important factors, and is therefore insufficient for adequate decision making. Consequently, in our research, we aim to model the high-level decision making of anesthesiologists, considering various factors, using supervised machine learning methods to make decisions at a higher level of abstraction than was done in previous research. Figure  1 shows the overall view of the proposed approach, which addresses the problem of predicting remifentanil increase events for unknown cases through supervised learning from data taken from existing cases. Predicting the increase in flow rate is a more critical task than predicting the decrease in flow rate because the increase in anesthetic coincides with the timing at which the anesthesiologist judges that the patient is in pain; therefore, we focus our prediction on the timing of the anesthetic increase. In our experiment, we compare the performance of six machine learning methods: logistic regression, support vector machine (SVM), random forest, LightGBM, ANN, and LSTM. We also apply Shapley additive explanations (SHAP), a machine learning model interpretation method—to their predictions, to examine the importance and contribution of the features, and obtain a qualitative understanding about anesthesiologist decision making.

## Results

### Performance comparison with different prediction horizons and time-series feature lengths

In this study, we address the binary classification problem of predicting remifentanil flow-increase events *n* min after each time point during surgery using general anesthesia from the patient’s basic information, vital signs, and drug histories. For a more specific setting, the patient’s basic information for a certain case and the vital signs and drug histories up to time *t* are given as observation data with the feature vector $\varvec{x}_{i}$ $(i=1,2,\dots ,t)$. We also treat $y_{t+1} \in \{0, 1\}$, which represents whether the flow rate of remifentanil increases at each of the time points $t+1,t+2,\dots ,t+n$, as the labels to be predicted. Here, $y_{t+1}=1$ indicates that the flow rate will increase within *n* min in the future, and $y_{t+1}=0$ indicates that the flow rate will not increase. To evaluate the performance, prediction results for unknown cases were evaluated with respect to accuracy, sensitivity, specificity, precision, and ROC-AUC. In the following experiments, the 30 min after the start of surgery and the 30 min before the end of the surgery are excluded from the time interval for prediction, as this is when the operation of the anesthesia is the most complicated. We used the LSTM model for prediction in the experiments, which are described in this section.

First, we analyzed the difficulty of predicting the problem by comparing the results of prediction performance in various settings with different time periods. We predicted the increase in the flow of remifentanil for five different time periods, ranging from 1 to 5 min in the future. Based on the results in Table 1, we observe that, when the prediction period was shorter, the performance was better for all indicators, except for the precision metric. In particular, for AUC, the best average performance was for the 1-min prediction. The performance became steadily worse when predicting for 2, 3, 4, and 5 min. To confirm that there was a difference in the mean values of AUC between 1- and 2-min predictions, 2- and 3-min predictions, 3- and 4-min predictions, and 4- and 5-min predictions, *t*-tests were performed. The *p*-values for these tests were $p\,<\,0.001$, $p\,<\,0.001$, $p\,=\,0.002$, and $p\,=\,0.023$, confirming statistically significant differences. These results demonstrate that 1-min predictions were the most accurate; when the prediction period was longer, it became more difficult to make predictions. As the time interval to be predicted became longer, the number of positive examples in the data increases, and thus the tolerance for the predicted values becomes wider. Alternatively, the classifier had to identify positive examples several minutes before the actual increase in flow was observed, which made the problem more difficult. In all of our subsequent experiments, we confined the problem to that of predicting an increase in flow within 1 min.

Next, we compared the prediction performance by changing the length of the time period of the time series data used as the vital features. As shown in Table 2, there was almost no difference in performance when the period used for prediction is changed between 3 and 5 min. In fact, when the *t*-test was conducted, the *p*-values for accuracy, sensitivity, specificity, precision, and AUC were 0.205, 0.622, 0.207, 0.454, and 0.932, respectively, with no significant difference in any of the indices.

**Table 1:** Here, we present results to compare prediction performance for different lengths of time intervals when predicting flux increases.

Prediction horizon (min)	Accuracy	Sensitivity	Specificity	Precision	AUC
1	**0**.**731**	**0**.**659**	**0**.**732**	0.023	**0**.**753**
2	0.706	0.620	0.708	0.040	0.713
3	0.689	0.590	0.692	0.053	0.687
4	0.678	0.560	0.683	0.065	0.667
5	0.667	0.546	0.673	**0**.**075**	0.652

Values in bold indicate the best score for the same indicator. With the exception of the precision metric, performance improved as the prediction time becomes shorter. We confirmed statistically significant differences in terms of AUC using *t*-tests among all problem settings.

**Table 2:** Comparison of the performance when the length of the time series feature used for prediction was set to either 3 or 5 min.

Time series feature length (min.)	Accuracy	Sensitivity	Specificity	Precision	AUC
3	0.725	0.663	0.725	0.023	0.753
5	0.734	0.650	0.734	0.023	0.753

For all evaluation metrics, the performance was similar, and no significant differences were observed using the *t*-tests.

### Comparison of the performance of different machine learning models

Table 3 presents the comparison of the predictive performance of the six different machine learning models: logistic regression, SVM, random forest, LightGBM, ANN, and LSTM. With respect to AUC, all the methods scored above 0.7; with respect to accuracy, sensitivity, and specificity, the evaluation values were relatively high, i.e., above 0.65, for all models except for SVM. Alternatively, the precision score was approximately 0.02, indicating that a certain number of false positives are inevitable to maintain high sensitivity. In this study, a false positive represents that the model incorrectly predicts an increase in remifentanil flow even though the anesthesiologist does not actually increase it. Based on these comparisons, the model using LSTM demonstrated the best overall predictive performance, with an AUC of 0.753. Additionally, our results show that even a basic linear model, such as logistic regression, can produce comparable performance. In summary, we can see that the choice of machine learning model does not have a critical effect on the results.

**Table 3:** Comparison of the different machine learning models. The choice of machine learning model did not have a critical impact on the results.

	Accuracy	Sensitivity	Specificity	Precision	AUC
Logistic regression	0.699	0.691	0.699	0.022	0.752
SVM	0.739	0.590	0.740	0.022	0.720
Random forest	0.749	0.563	0.751	0.022	0.713
LightGBM	0.705	0.659	0.706	0.021	0.738
ANN	0.642	0.739	0.641	0.020	0.742
LSTM	0.731	0.659	0.732	0.023	0.753

### Analysis of the contributions of features to prediction models and their predictions

To analyze the results in more detail, we examined the contribution of each feature to an entire model in addition to the prediction for each data instance.

For each model, we examined the contribution of each feature to the entire model using the mean absolute SHAP value. We used three non-time-series features: timestamp, remifentanil flow, and one-shot elapsed time as well as four basic features, patient’s gender, age, weight, and height. We also used seven time-series features: hear rate (HR), systolic blood pressure (SBP), mean arterial pressure (MAP), diastolic blood pressure (DBP), respiratory rate (RR), oxygen saturation (SpO2), and end tidal carbon dioxide (EtCO2). These were used for the past 3 min, which resulted in 21 features in total.

**Figure 2:**
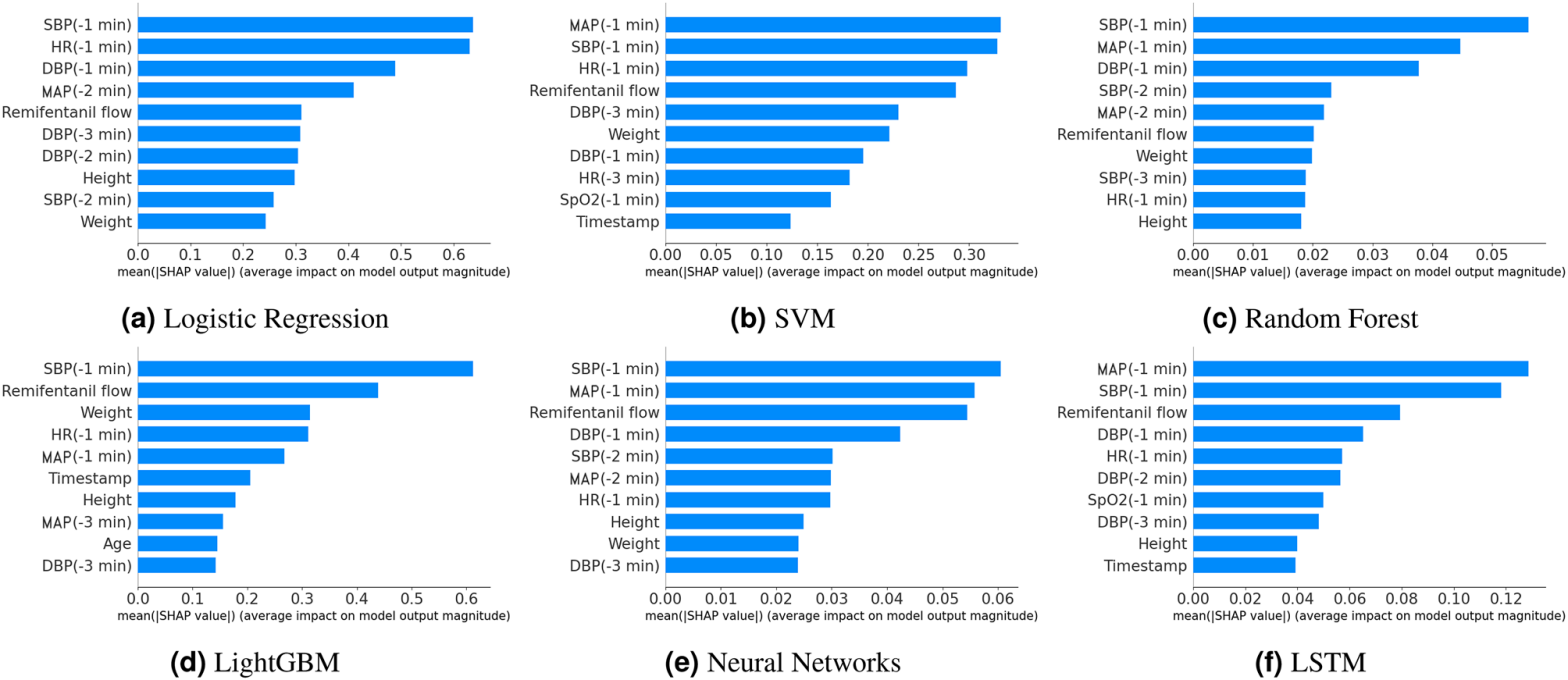
Top 10 important features calculated according to mean absolute SHAP values. SBP, MAP, HR, remifentanil flow, and patient weight immediately before prediction are relatively important features for all methods.

Figure 2 shows the top 10 features in terms of contribution among the total 28 features. Among the features related to blood pressure, the importance of SBP was particularly high; i.e., it was the first or second most important feature among all the model. MAP was also of high importance in all the models, although not as high as SBP; alternatively, DBP was relatively unimportant. Next, HR and remifentanil flow rate were relatively high in importance. Among the basic patient information, weight was important in some models, but these basic factors, including age, were not particularly important for prediction. Additionally, the features regarding respiration, such as RR, SpO2, and EtCO2, were not considered important. Vital values at the time just before the prediction tended to be more important than the older values. This suggests that the model captured the habit of anesthesiologists, who often make decisions based on the most recent information.

**Figure 3:**
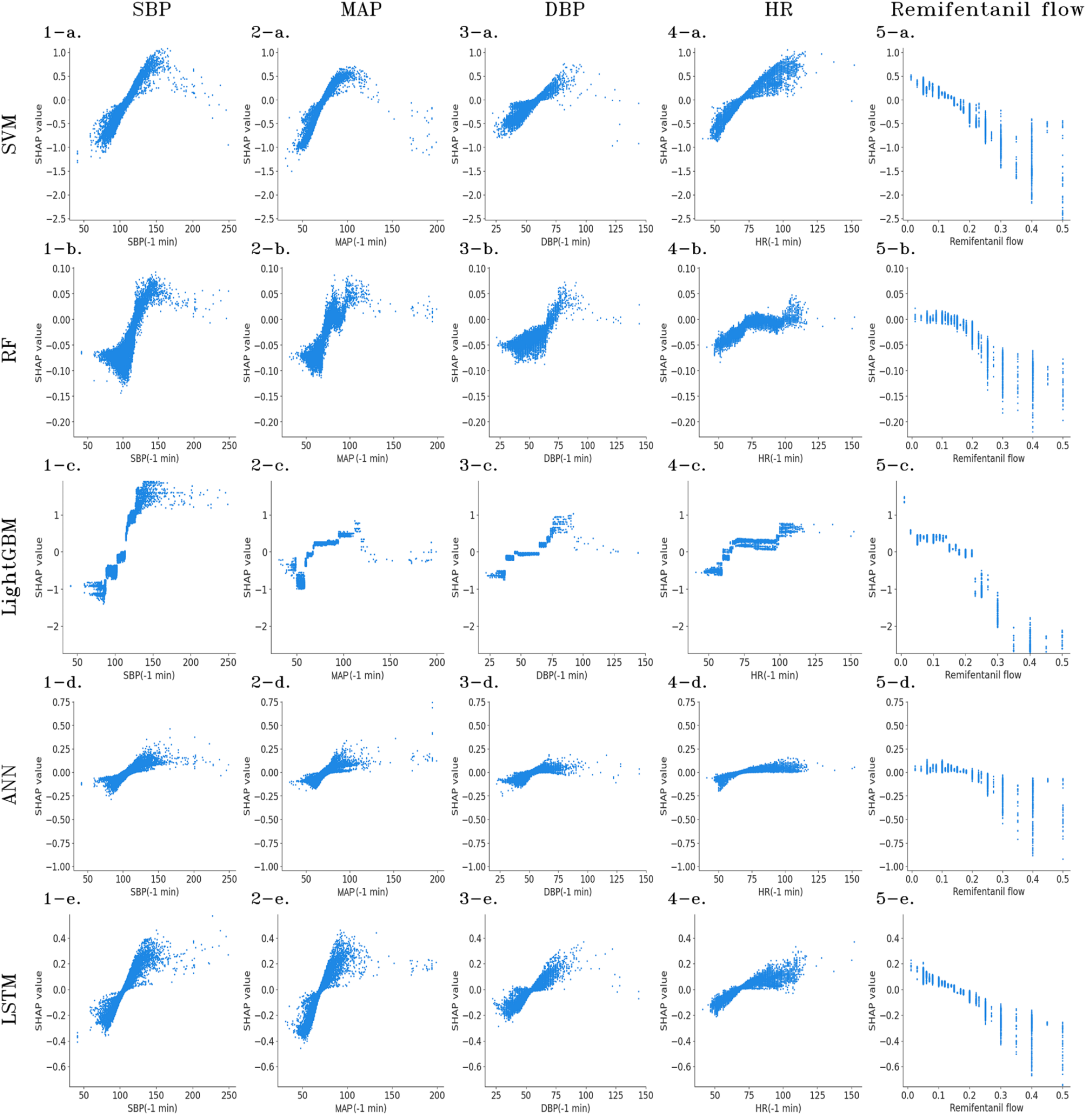
Contribution of each feature to individual predictions. The x-axis represents the value of the feature, and the y-axis represents the SHAP value. Positive SHAP values indicate the model tends to predict that the flow will increase, whereas negative values indicate that the flow will not increase. BP and HR show correlations with SHAP values, whereas remifentanil flow shows negative correlations.

For each of the features of particular importance in the aforementioned analysis, which namely, SBP, MAP, DBP, HR, and remifentanil flows immediately before the prediction, we analyzed the contribution of each to the prediction results. In the scatter plots in Fig. 3, the x-axis represents the value of the feature and the y-axis represents the SHAP value. If the SHAP value was positive, the model tended to predict that the flow would increase; conversely, if the value was negative, the model tended to predict that the flow would not increase. Note that, because logistic regression is a linear model, the points are plotted on a straight line, and therefore, the results are omitted.

For SBP, there was a positive correlation between the vital and SHAP values. Furthermore, considering the value at which the positive and negative SHAP values switch, the threshold value for all models was approximately 100–125 mmHg. A scatter plot with a similar shape is drawn for MAP, and the boundary between the positive and negative SHAP values was approximately 70–90 mmHg. A similarly shaped scatter plot is drawn for DBP; however, the variance of SHAP values was smaller than that of SBP and MAP, indicating that the influence of SHAP was small, as discussed in the previous analysis. Furthermore, the range of values for DBP that resulted in values near the positive and negative boundaries of SHAP was wide (approximately 40–75 mmHg), indicating that there was ambiguity in predictions based on DBP. For HR, there was a positive correlation between vital and SHAP values; however, it was not as strong as the values for blood pressure. In addition, the range of values that served as the boundary between the positive and negative SHAP values was as wide as 60–100 bpm, and the SHAP values tended to take on large values when they exceeded 100 bpm; this trend was particularly pronounced for LightGBM. Regarding remifentanil flow, there was a negative correlation between the flow and SHAP value; although there was a wide flow range where the SHAP value was zero, it tended to predict no increase when the flow exceeded 0.3 $\upmu$g/ml/min. The SHAP value did not take large positive values, and instead, tended to take large absolute values for negative values indicating that remifentanil flow contributed to predicting no increase in flow.

## Discussion

In this study, we addressed the problem of predicting future increase in remifentanil flow rates that correspond to intraoperative decision making by anesthesiologists, based on basic patient information as well as past vital signs and drug use history. Specifically, we compared the prediction results in several problem settings with different time horizons to be predicted, observed that the 1 min time windows worked best for predicting future incremental events, and verified the prediction performance using six machine learning models. Furthermore, to interpret the obtained prediction models and verify the validity of the predictions, we conducted an analysis using SHAP values—a prediction interpretation method for machine learning prediction models.

By comparing problem settings with different time periods for forecasting, we found that the shorter the time period, the higher the forecasting performance. Alternatively, the comparison of different periods of time series features used for forecasting showed that relatively old data, namely that which are far from the time of forecasting, are not significant for making predictions. From the above observations, it can be understood that the most recent data are more important in the decision making of anesthesiologists.

Next, we discuss the performance comparison of different machine learning models. We conducted a comparison experiment using six models: Logistic regression, SVM, random forest, LightGBM, ANN, and LSTM. The results showed that LSTM, which is specialized for time series data, had the best performance, with an accuracy of 0.728, a sensitivity of 0.664, a specificity of 0.729, a precision of 0.023, and an AUC of 0.753. However, even a simple and computationally inexpensive linear model such as logistic regression showed prediction performance comparable to LSTM which is explicitly encodes feature history. One of the reasons is that the latest feature values are significant for prediction, and all other models also gave the feature history as input. Another reason is that the length of the input series used for the features was short, up to 5 minutes, so we could not take advantage of the LSTM ability to preserve long-term dependencies. The lack of significant differences in prediction performance between the different machine learning models suggests that the predictability in the prediction problem addressed in this study is stable.

Next, we analyzed the contribution of the features to the model and its predictions using SHAP—a method for interpreting the predictions of machine learning models. In terms of the contribution of features to the model, SBP was found to be particularly important, contributing more than MAP on average. In addition to the blood pressure data, the HR and immediately preceding remifentanil flow rate data were also found to be important. In our preliminary assumptions, we speculated that HR was as important as blood pressure. However, it was less important than blood pressure on average; this may be because the prediction targets in this study were only increased anesthetic events. In general, we would imagine that anesthetists would lower the flow rate of remifentanil when the HR is low; however, for increased flow, it is possible that it contributed to the prediction only when the HR was extremely high. In addition, the age, gender, height, and weight of patients were included in the features; however, with the exception of weight, they did not contribute to the prediction as much as the vital values. This result can be explained in a similar way to the previous case of HR. That is, the patient’s age did not contribute as much to the prediction of the increased flow events, although we can imagine that it is related to the drug dosage.

In addition, we examined the validity of the models by comparing the trends of the models found by visualizing SHAP values with clinical knowledge. For blood pressure, there is clinical knowledge that it is recommended to maintain $\mathrm{SBP}\ge {100} \,{\hbox {mmHg}}$ and $\mathrm{MAP}\ge {65}\,\mathrm{mmHg}$. It is also preferred to set targets within a range of up to 20% of baseline blood pressure^23^. Other research states that, if the standard baseline BP is 90–129 mmHg for SBP and 50–79 mmHg for DBP, the target BP should be within 10% of the baseline BP and 65–95 mmHg for MAP^24^. In our experiments, the values of blood pressure at which the positive and negative SHAP values switched were approximately 100–125 mmHg for SBP and 70–90 mmHg for MAP, indicating that the prediction models were making reasonable judgments because the values of blood pressure that do not contribute specifically to prediction were included in the range of target values. Next, for HR, clinical knowledge indicates that it is appropriate to keep $\mathrm{HR}<{100} \,\mathrm{bpm}$^23^. With respect to the model, the probability of predicting an increase in the flow rate of remifentanil was also increased once the 100 mmHg was exceeded. By analyzing the importance of the features and the contribution of each feature, we found that the model had a predictive tendency that was partially consistent with the clinical findings of anesthesiologists.

Notably, there are some limitations to our study. The first limitation is that the precision of the prediction is not very high. The reason for this low precision was that we focused on sensitivity and specificity in solving a difficult problem with extremely unbalanced data labels, and adjusted the model so that these values would be high. The second limitation is that the anesthetic to be predicted in the experiment is limited to the analgesic remifentanil, making the model impractical. Anesthetics include not only analgesics but also sedatives and muscle relaxants, which must be used in combination to keep the patient in the best possible condition during surgery. In this study, we could not obtain sufficient data for anesthetics other than remifentanil, and thus the experiment could not be conducted. However, before considering the actual application of the model, it is worthwhile to increase the amount of data and validate the predictions for multiple drugs to improve the model’s practicality. The third limitation is that the prediction was limited to flow increase events. Predicting the increase of anesthetics is a critical task, but it is necessary to predict the decrease as well as the increase event. This is because there is an upper limit to the amount of anesthetic that can be used during surgery, so predicting increases alone is not practical. We should be aware that increasing the dose of anesthetics also has risks depending on the patient’s information, such as age and weight. In addition, complete control of anesthesia will eventually require extending the prediction to the flow rate of the anesthetic itself.

In conclusion, this study attempted to predict events in the anesthesia administration during general anesthesia, particularly the increase in flow rate during continuous remifentanil administration, formulated as a binary classification problem and solved using supervised learning. The importance of recent data in prediction was confirmed by examining several problem settings and the analysis of features. With respect to prediction performance, although the model using LSTM achieved the best performance, the fact that the performance was comparable to all of the other machine learning methods showed the stable predictability of this task. Furthermore, the interpretation of the obtained models using SHAP—a machine learning model interpretation method was consistent with medical knowledge in anesthesiologists’ decision making.

## Materials and methods

### Data source

This study used electronic anesthesia record data of patients who underwent surgery and had anesthesia records at Okayama University Hospital. The data collection period was one month, from October 1, 2018 to October 31, 2018, and 449 records were collected based on the following criteria: (1) patients whose medications included anesthetics, analgesics, muscle relaxants, or vasoactive agents; (2) patients who received general anesthesia; (3) patients whose age was 20 years or older; and (4) patients who did not use artificial heart or lungs.

The data comprised three types of records: patient information, vital records, and drug records. The patient information include four types of information: age, weight, height, and gender. These were processed in such a way that the individual could not be identified. The vital records were time-series data observed every minute and contained 23 types of features, seven of which (HR, SBP, MAP, DBP, RR, SpO2, and EtCO2) were used when considering the percentage of missing data. Drug records, also in time-series format, were observed every minute and recorded as the history of drug use. In addition to the time and dose used, the record contains information indicating whether the administration method was through one-shot or continuous administration. In this study, we employed the usage history of one-shot doses of remifentanil and fentanyl, which have similar effects to remifentanil, as the prediction targets. The features created from the vital and drug records are shown in Table 4.

**Table 4:** Details of collected data variables.

Variable	Description
Timestamp (min)	Elapsed time in minutes
HR (bpm)	Heart rate per minute
SBP (mmHg)	Systolic blood pressure
MAP (mmHg)	Mean arterial pressure
DBP (mmHg)	Diastolic blood pressure
RR (breaths per minute)	Respiratory rate
SpO2 (%)	Oxygen saturation
EtCO2 (%)	End tidal carbon dioxide
Remifentanil flow ($\upmu$ g/ml/min)	Remifentanil flow rate
One-shot elasped time (min)	Elapsed minutes since the last observed Remifentanil/fentanyl one-shot infusion

### Data selection and statistical methods

**Figure 4:**
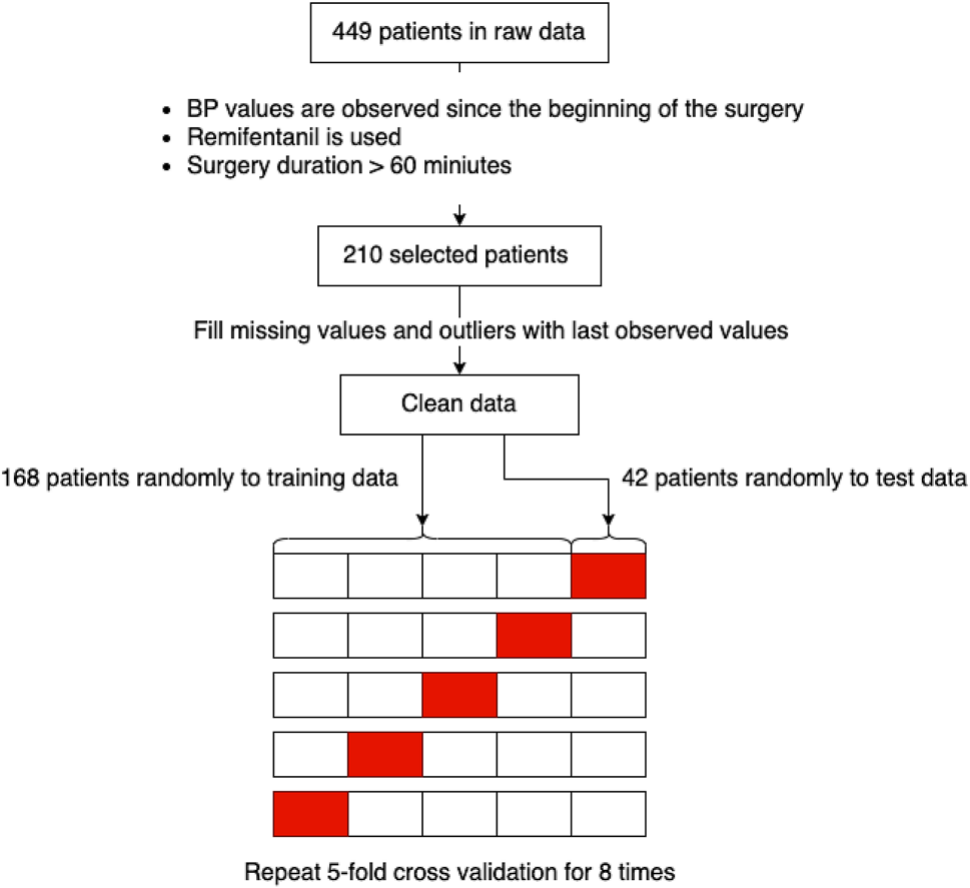
Flowchart for data process. In the 210 cases selected by multiple criteria, the data were preprocessed, and then, randomly divided into five parts for cross-validation.

The collected data were selected and preprocessed according to the flow shown in Fig. 4. We focused on cases that met the following three criteria: (1) blood pressure was observed from the beginning of the surgery; (2) remifentanil was used; and (3) the duration of the surgery was more than 60 min, resulting in 210 cases, as shown in Table 5. Excluding the 30 min of data immediately after the start and just before the end of the surgery, the total data was 42,075 min. The percentage of labels present in the entire data was 405 (0.9625% of the total) for positive cases and 41,670 (99.0374% of the total) for negative cases.

**Table 5:** Statistics of the selected 210 patients data.

Item	Total	Male	Female
Sex ratio	210	103 (49.0%)	107 (51.0%)
Age (mean±sd)	$62.56 \pm 15.48$ years	$63.56 \pm 14.76$ years	$61.59 \pm 16.07$ years
Height (mean±sd)	$160.08 \pm 9.55$ cm	$166.97 \pm 6.81$ cm	$153.58 \pm 6.82$ cm
Weight (mean±sd)	$58.96 \pm 12.75$ kg	$64.17 \pm 11.39$ kg	$54.05 \pm 11.99$ kg

Missing values and outliers in the data were complemented using the previously observed values. Outliers were determined on a rule basis, and outliers were defined as data that did not satisfy one of the following criteria: $40<\text {SBP}<250$, $30<\text {MAP}<200$, and $20<\text {DBP}<200$. Cross-validation was used in all experiments to verify the prediction performance. We used repeated 5-fold cross validation, in which the splits were randomly recreated, and the average of their performance was calculated. The number of iterations of cross-validation was set to 8, and the mean of the results for 40 samples is presented in this paper. For the *t*-tests, a significant difference was considered to exist when the *p*-value was 0.05 or less. The decision threshold for performance verification with test data was approximated to be the value that minimized the difference between sensitivity and specificity in prediction with training data.

### Machine learning models and training

In this study, we used six models for the experiments: logistic regression, SVM, random forest, LightGBM, ANN, and LSTM.

Logistic regression is a stochastic linear binary classification model that is used in several fields. The quasi-Newton method is used as the optimization algorithm for the objective function. SVM is a classifier based on the margin maximization principle, and can perform nonlinear classification using kernel functions. In this study, we used the RBF kernel. Random forest learns a model that comprises an ensemble of multiple decision trees. In this study, the number of trees was set to 100. LightGBM, which is also an ensemble model of multiple decision trees, is based on the concept of gradient boosting; it sequentially adds decision trees^25^. The Python library, Optuna, was used to tune the hyperparameters, and each parameter was determined in a step-wise manner^26^.

An ANN is a multilayer perceptron that can approximate complex functions through multiple layers of nonlinear transformations (activation functions). In this study, we used three hidden layers and the ReLU function as its activation function. The Adam optimizer was used as the optimization algorithm. To prevent overfitting, we used dropout. In the final layer, a sigmoid function was used to obtain a value between 0 and 1, which was treated as a probabilistic output.

**Figure 5:**
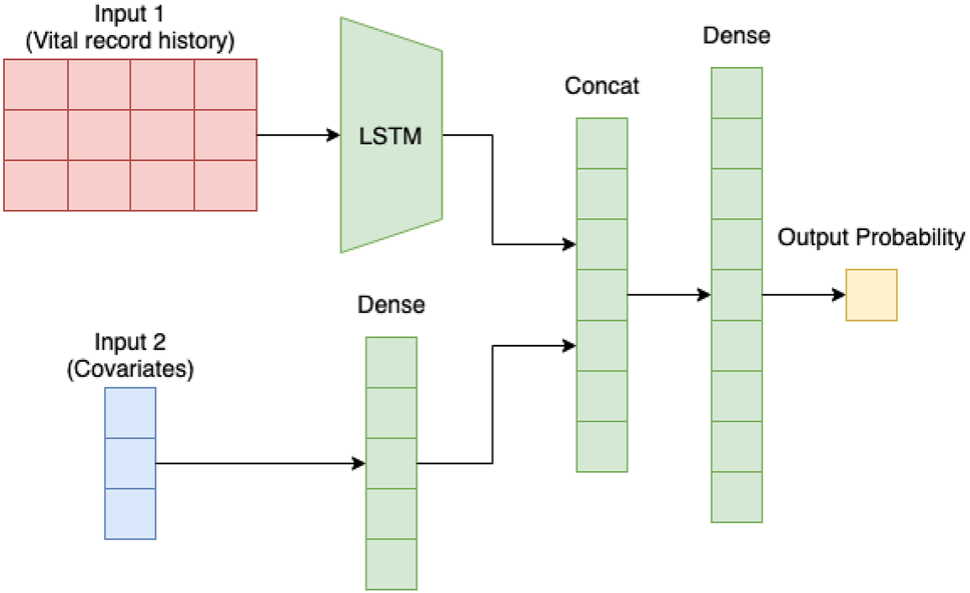
LSTM model architecture used in our experiments.

LSTM is a type of RNN, which is a widely used ANN to model time series data^27^. In this study, we used the architecture that is shown in Fig. 5. As the dataset used in this study contains both time-series data and non-time-series data in the input, the time-series data were given to the LSTM block, and the non-time-series data were given as input to the fully connected layer. Then, the outputs from these blocks were concatenated and input to the fully connected layer again; the final output was obtained as a probabilistic value between 0 and 1 by the sigmoid function. As in a normal ANN, the ReLU function was used as the activation function in the fully connected layer, and Adam was used for optimization. To prevent overfitting, L2 regularization of weights and dropout after the fully connected layer were applied.

For models that required hyperparameter tuning during training, 20% of the training data was used as validation data for tuning. In all the models, to handle with data imbalances, each data instance was weighted by the inverse of the class ratio during training, so that positive examples, which are the minority in this case, had a larger influence on the training of the model^28^. In addition, SVM, random forest, and ANN sampled the negative examples so that the positive examples would be 10% of the total by random undersampling to make the training proceed successfully^29^. In addition, we used bagging, i.e., multiple sampled data sets were created, multiple models were trained, and an ensemble of these models was used to increase the performance and stability of the predictions^30^. The final prediction result is the average of the prediction probabilities of the multiple models using soft voting.

### Interpretable machine learning models

To add interpretability to our machine learning models, we used SHAP values^31^. A SHAP value is the contribution of each feature to the predicted outcome of a model, based on cooperative game theory, and is a method often used to interpret the predictions of machine learning models. Because we handle a binary classification problem in this study, a positive SHAP value indicates that the feature contributes to the model prediction where the flow rate of remifentanil will be increased, whereas a negative SHAP value indicates that the feature contributes to the model prediction where the rate will not be increased. We plotted the SHAP values for each feature to understand the trend of the model prediction. The contribution of a feature to the overall model was obtained by averaging the absolute values of the SHAP values for all the data.

### Ethical approval

This study protocol was approved by the Ethics Review Committee of Okayama University (protocol no. K1910-028), and the research was conducted in compliance with the Declaration of Helsinki and the Ethical Guidelines for Medical and Biological Research Involving Human Subjects. Informed consent was obtained in the form of opt-out on the website. Those who rejected were excluded.
